# Hyperglycaemia and *Pseudomonas aeruginosa* acidify cystic fibrosis airway surface liquid by elevating epithelial monocarboxylate transporter 2 dependent lactate-H^+^ secretion

**DOI:** 10.1038/srep37955

**Published:** 2016-11-29

**Authors:** James Peter Garnett, Kameljit K. Kalsi, Mirko Sobotta, Jade Bearham, Georgina Carr, Jason Powell, Malcolm Brodlie, Christopher Ward, Robert Tarran, Deborah L. Baines

**Affiliations:** 1 Institute of Cellular Medicine, Newcastle University, Newcastle-upon-Tyne, UK; 2 Immunology & Respiratory Diseases Research, Boehringer Ingelheim Pharma GmbH & Co. KG, Biberach an der Riss, Germany; 3 Institute for Infection and Immunity, St George’s, University of London, Tooting, London, UK; 4 Cystic Fibrosis Centre/Marisco Lung Institute, University of North Carolina, Chapel Hill, NC, USA

## Abstract

The cystic fibrosis (CF) airway surface liquid (ASL) provides a nutrient rich environment for bacterial growth including elevated glucose, which together with defective bacterial killing due to aberrant HCO_3_^−^ transport and acidic ASL, make the CF airways susceptible to colonisation by respiratory pathogens such as *Pseudomonas aeruginosa*. Approximately half of adults with CF have CF related diabetes (CFRD) and this is associated with increased respiratory decline. CF ASL contains elevated lactate concentrations and hyperglycaemia can also increase ASL lactate. We show that primary human bronchial epithelial (HBE) cells secrete lactate into ASL, which is elevated in hyperglycaemia. This leads to ASL acidification in CFHBE, which could only be mimicked in non-CF HBE following HCO_3_^−^ removal. Hyperglycaemia-induced changes in ASL lactate and pH were exacerbated by the presence of *P. aeruginosa* and were attenuated by inhibition of monocarboxylate lactate-H^+^ co-transporters (MCTs) with AR-C155858. We conclude that hyperglycaemia and *P. aeruginosa* induce a metabolic shift which increases lactate generation and efflux into ASL via epithelial MCT2 transporters. Normal airways compensate for MCT-driven H^+^ secretion by secreting HCO_3_^−^, a process which is dysfunctional in CF airway epithelium leading to ASL acidification and that these processes may contribute to worsening respiratory disease in CFRD.

In people with cystic fibrosis (CF), the microbiocidal activity of the airway surface liquid (ASL) is defective due to aberrant HCO_3_^−^ transport and acidic pH^1^,^2^,^3^ and there is a nutrient rich environment for bacterial growth including elevated concentrations of amino acids, mucins, iron and glucose^4^. In CF, coexisting diabetes mellitus (CF-related diabetes; CFRD) is associated with an increased risk of infection with multiple antibiotic-resistant *P. aeruginosa*. Patients with CFRD have more pulmonary exacerbations and respond less well to intravenous antibiotics, than those without diabetes^5^. In humans, we have shown that ASL glucose concentrations are normally lower than those of plasma (0.4 mM compared to 5 mM) but are elevated in patients with CF (~2 mM) and are further elevated in CFRD (~4 mM)^6^. In intubated patients on intensive care, elevated glucose concentrations in bronchial aspirates were associated with the presence and acquisition of respiratory pathogens^7^. In human airway epithelial cells grown at air-liquid interface, elevation of basolateral glucose concentrations increased ASL glucose concentrations and promoted the growth of respiratory pathogens such as *P. aeruginosa* over and above the effects of other hallmarks of the CF ASL including acidic pH and mucus hyperviscosity^8^,^9^.

In CF sputum, lactate concentrations are also elevated, which is thought to be predominately caused by invading neutrophils^10^. Cellular lactate production by cancer cells in aerobic conditions (the Warburg effect) is well documented^11^. However, glycolysis progression to lactate production under aerobic conditions is a normal feature of mammalian cell metabolism, even in tissues that take up lactate from the circulation, such as the heart^12^. Therefore, it is conceivable that airway epithelial cells contribute to the elevated ASL lactate concentrations seen in CF and CFRD. Mammalian cells move lactate, in part, through monocarboxylate transporters (MCT) which co-transport lactate with H^+^^13^. Thus, epithelial lactate secretion would be predicted to acidify the extracellular fluid, a known feature of the CF ASL.

We therefore tested the hypothesis that airway epithelial lactate production and ASL lactate concentration was elevated in response to hyperglycaemia in CF and non-CF primary cultured human airway epithelial cells (HBE) and cell lines H441 and Calu-3. We determined whether epithelial lactate production contributed to the acidic ASL observed in CF by secretion via MCTs. In addition, due to the prevalence of *P. aeruginosa* infections in CFRD, we investigated the influence of the bacterium on epithelial lactate production and ASL pH.

## Materials and Methods

### Airway epithelial cell culture

Primary human bronchial epithelial (HBE) cells (non-CF and CF) were obtained from endobronchial brushings or extracted from explanted lungs and cultured as previously described^14^,^15^. HBE cells were obtained with informed written consent from all study patients and in accordance with approval from the Newcastle and North Tyneside Local Regional Ethics Committee (reference number 2001/179 and 07/Q0906/47) The CF Center Tissue Core, University of North Carolina at Chapel Hill Biomedical Institutional Review Board (protocol #03-1396). Cells were transferred onto clear Transwell^®^ (Costar) inserts (1.12 cm^2^ area, 0.45-μm pore size) and grown at air-liquid-interface (ALI) to form confluent fully differentiated monolayers. Cells were studied 3–5 weeks post-seeding.

The human airway epithelial cell lines, Calu-3 and H441 (from ATCC), were cultured and grown on Transwell^®^ inserts to form confluent differentiated monolayers as previously described^9^,^16^. Experiments were carried out 10–14 days post-seeding.

### Airway surface liquid (ASL) glucose and lactate measurements

ASL glucose and lactate concentrations were assessed by washing the apical surface with 50 μl of Krebs salt solution and analysed using enzymatic glucose and lactate assay kits (Sigma, UK). ASL glucose and lactate concentrations were calculated assuming an original ASL volume of ~1 μl (except Calu-3 cultures in which ASL volume was measured by pipette). Normal blood glucose or hyperglycaemia was modelled by applying D-glucose concentrations of 5 or 15 mM to the basolateral Krebs salt solution, respectively. 10 mM L-glucose (non-metabolisable stereoisomer of D-glucose) was added to 5 mM D-glucose solution to maintain osmotic balance between solutions. MCT1/2 inhibition was achieved using the specific inhibitor AR-C155858^17^. The presence of other functional MCT was investigated using phloretin (50 μM), a concentration known to inhibit several MCTs but not glucose uptake via GLUT transporters^18^.

### Seahorse Glycolysis Stress Assay

20,000 Calu-3 cells per well were seeded into a Seahorse XF96 plate and incubated at 37 °C, 5% CO2 for 48 hours. The medium was changed 24 hours prior to Seahorse experiment with or without 100 μg/mL LPS. Three days post-seeding, the Seahorse Glycolysis Stress Assay was performed according to the manufacturer’s instructions with the injection of 5 or 15 mM Glucose. The plate layout was separated into quadrants to reduce edge effects.

For LPS experiments, viable cells were quantified after the seahorse assay using CyQuant Direct according to the manufacturer’s instructions. Briefly, the cells’ supernatant was partly aspirated to reach a volume of 100 μL, then 100 μL of the freshly prepared staining solution was added to the cells. After incubation for 1 h at 37 °C without CO2, fluorescence was read with bottom reading mode of a standard plate reader.

### FRET intracellular lactate measurements

H441 cells grown on polylysine coated coverslips (NeuVitro, Germany) were transiently transfected with Laconic (a kind gift of Prof P. Barros, Centro de Estudios Científicos, Chile^19^, using Lipofectamine 2000 (Thermo Fisher, UK). Cells were imaged 24–48 hours post transfection at 37 °C in a 95% air, 5% CO_2_ gassed Krebs salt solution, supplemented with glucose and/or inhibitors (as above), using a Zeiss LSM 510 Meta confocal microscope with a 20x Pan-Neofluar lens. Laconic is labelled with fluorophores mTFP and Venus and reports a reduction in FRET efficiency (%E) with lactate binding. This was measured by analysing the fluorescence emission of mTFP (donor) and Venus (acceptor) in a selected area within a cell after excitation at 458 nm. The Venus (acceptor) fluorescence within the selected area was photo bleached (>80%) by excitation at 514 nm using full laser capacity and the fluorescence emission of both fluorophores re-measured. The FRET efficiency (%E) was then calculated. FRET ratio was measured using rapid excitation at 458 nm and recording emission at 475–525 nm (mTFP) and 530–600 nm (Venus) every second over a time course of 10 minutes. Elevation of intracellular lactate results in an increase in FRET ratio (mTFP/Venus).

### MCT immunocytochemistry

Cells grown on Transwell^®^ inserts were initially fixed with methanol:acetone for 10 min at room temperature, washed with PBS followed by apical membrane biotinylation with biotin anti-goat IgG (1:200 dilution) and labelled with streptavidin conjugated to Alexa Fluor 594 (red) or 488 (green) (1:200 dilution). The apical surface was washed with PBS and cells blocked and permeabilized with 1% bovine serum albumin containing 0.1% Triton in Tris-buffered saline. Primary antibodies for MCT1 (sc-365501) and MCT4 (sc-50329), were from Santa Cruz, USA. MCT2 (ab198272) and MCT3 (ab60333), were from AbCam, UK. Cells were incubated with 1:100 dilution of antiserum and incubated at room temperature for 1 hour. Cells were washed with PBS and the secondary antibody was added (anti-mouse Texas Red or anti-rabbit Alexa Fluor 488 at 1:100 dilution) for 30 min at room temperature. After rinsing with PBS, Transwell^®^ inserts were removed with a scalpel and mounted onto slides with Vectashield mounting medium containing 4′,6-diamino-2-phenylindole (DAPI) for nuclei staining. Images were visualised using a Zeiss LSM 510 Meta confocal microscope.

### Western blotting for MCT proteins

Cells were lysed in buffer containing 20 mM Tris-HCL pH 7.5, 150 mM NaCl, 1 mM EDTA, 50 mM NaF, 1 mM NaVO4, 1% w/v Triton-X, 0.5% w/v sodium deoxycholate, 0.1% w/v SDS, 1 μg/ml protease inhibitor cocktail (P8340, Sigma, UK). Cells were sonicated and cell debris was removed by centrifugation at 10,000 RPM at 4 °C for 10 mins. Total protein concentration was determined by Bradford Assay and between 30 μg–60 μg was electrophoresed on NuPage Novex 10% Bis-Tris Protein Gels (Invitrogen). Protein was then transferred onto polvinylidene difluoride membranes (Merck Millipore). Nonspecific reactivity was blocked in Odyssey Blocking Buffer (Licor, UK) diluted 1:1 in PBS for 1 h at room temperature. Blots were incubated overnight with primary antibodies described aboveMCT1 (1:1000 dilution), MCT2 (1:200 dilution) and MCT4 (1:1000 dilution). The blots were then washed in Tris-buffered saline/0.2% Tween 20 and incubated with either donkey anti-mouse IgG (926–3221) or donkey anti-rabbit IgG (Licor, UK) (both 1:40000 dilution) for 1 hr at room temperature. Blots were subsequently immunostained with a mouse monoclonal antibody to β-actin. Detection of antigen-antibody complexes was assessed using a Licor Odyssey western blot imaging system.

### ASL pH measurements

ASL pH of Calu-3 and H441 monolayers was monitored *in situ* using a pH microelectrode (Lazar, USA). 100 μl of Krebs salt solution was added to the apical surface of the monolayers at the start of the experiment to provide sufficient depth for the microelectrode to accurately measure pH. D-glucose concentrations of 5 or 15 mM were added the basolateral Krebs salt solution with or without inhibitors (as described above). To avoid addition of excess fluid, pH-sensitive dye was used to assess ASL pH across HBE and CF HBE monolayers: pH-sensitive pHrodo dextran (10 μM) and pH-insensitive Alexa 647 dextran (10 μM) were added to the Krebs salt solution and measured *in situ* on airway cultures using a Tecan Infinite M1000 plate reader at 37 °C, as previously described^3^. Due to auto-fluorescence of PA01, a pH microelectrode was used to measure ASL pH in bacteria-epithelia co-cultures.

### Airway epithelia - Pseudomonas aeruginosa co-culture

A single colony of *P. aeruginosa* PA01 was incubated overnight at 37 °C in EMEM (containing 5 mM glucose). Bacterial cultures were diluted in glucose-free Krebs salt solution (as above). 1 × 10^6^ CFU/ml PA01 was applied to the apical surface of HBE cell monolayers in 100 μl of solution. The basolateral side of the airway epithelial-bacterial co-cultures were washed and placed in Krebs salt solution 24 hours prior to co-culture experiments to remove antibiotics and components of the media that could influence bacterial growth. D-glucose concentrations of 5 or 15 mM were then added to the basolateral solution as described above. Co-cultures were placed in a CO_2_ incubator at 37 °C for 6 hours, after which ASL pH was monitored using a pH microelectrode and each was homogenised for CFU calculation by plating out serial dilutions on Luria agar.

### Chemicals and reagents

All chemicals and reagents were obtained from Sigma, Poole, UK unless otherwise stated.

### Statistical analysis

Values are reported as mean ± S.E.M. Statistical analysis was performed using analysis of variance (ANOVA) and post hoc Bonferroni multiple comparison or Student’s *t* test. *P* values < 0.05 were considered significant.

## Results

### Hyperglycaemia elevates glucose and lactate concentrations in airway surface liquid under aerobic culture conditions

**Figure 1:**
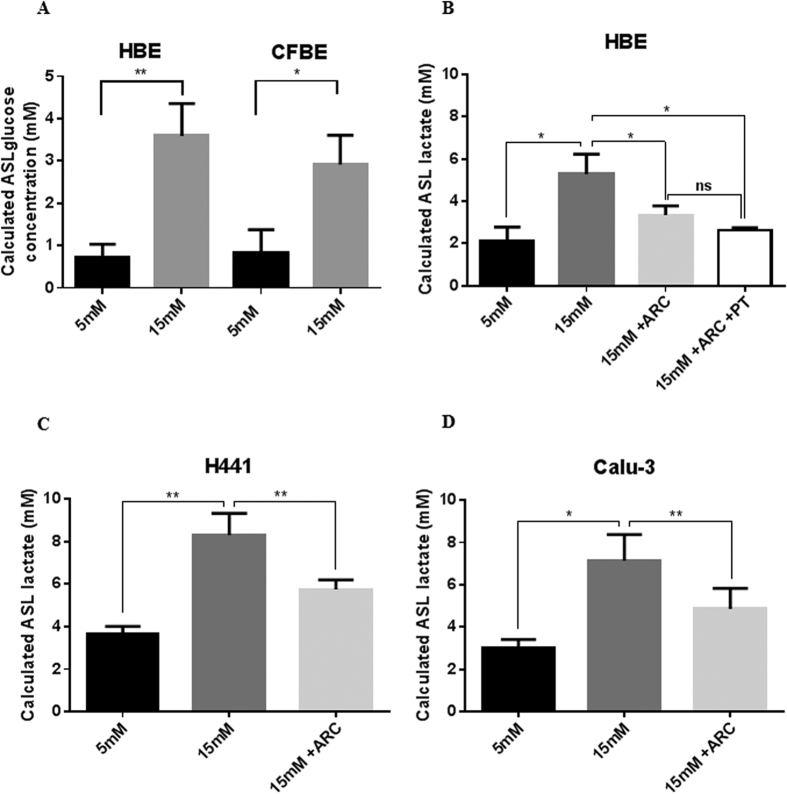
Hyperglycaemia increases airway surface liquid (ASL) glucose and monocarboxylate transporter (MCT)-dependent concentrations in primary airway epithelial cultures. (**A**) Glucose concentration of ASL from non-CF and CF primary HBE cell monolayers after 6 hours in the presence of basolateral 5 mM or 15 mM D-glucose. Lactate concentration of ASL from primary HBE cell (**B**) H441 (**C**) and Calu-3 (**D**) monolayers after 6 hours in the presence of basolateral 5 mM or 15 mM D-glucose in the absence or presence of 100 nM MCT2 inhibitor AR-C15585 (ARC) and 50 μM phloretin (PT). *ns* P > 0.05, *P < 0.05, **P < 0.01, n = 5–13. Data represent the means ± S.E.M.

Hyperglycaemia was mimicked in airway epithelial monolayers grown at air liquid interface by increasing D-glucose from 5 to 15 mM in the basolateral salt solution for 6 hours. This resulted in a significant rise in ASL glucose concentration in non-CF HBE and CFBE consistent with previous results in H441 and Calu3 cells^9^,^20^ (Fig. 1A). ASL lactate concentrations were elevated in all cell types (Fig. 1B–D) (2.1 ± 0.7 mM to 5.7 ± 1.5 mM in non-CF HBE monolayers; 3.7 ± 0.3 mM to 8.3 ± 1.0 mM in H441; 2.2 ± 0.7 mM to 7.1 ± 1.2 mM in Calu-3; P < 0.05, n = 7–13).

### Hyperglycaemia modifies glycolysis and elevates intracellular lactate concentrations in airway epithelial cells

**Figure 2:**
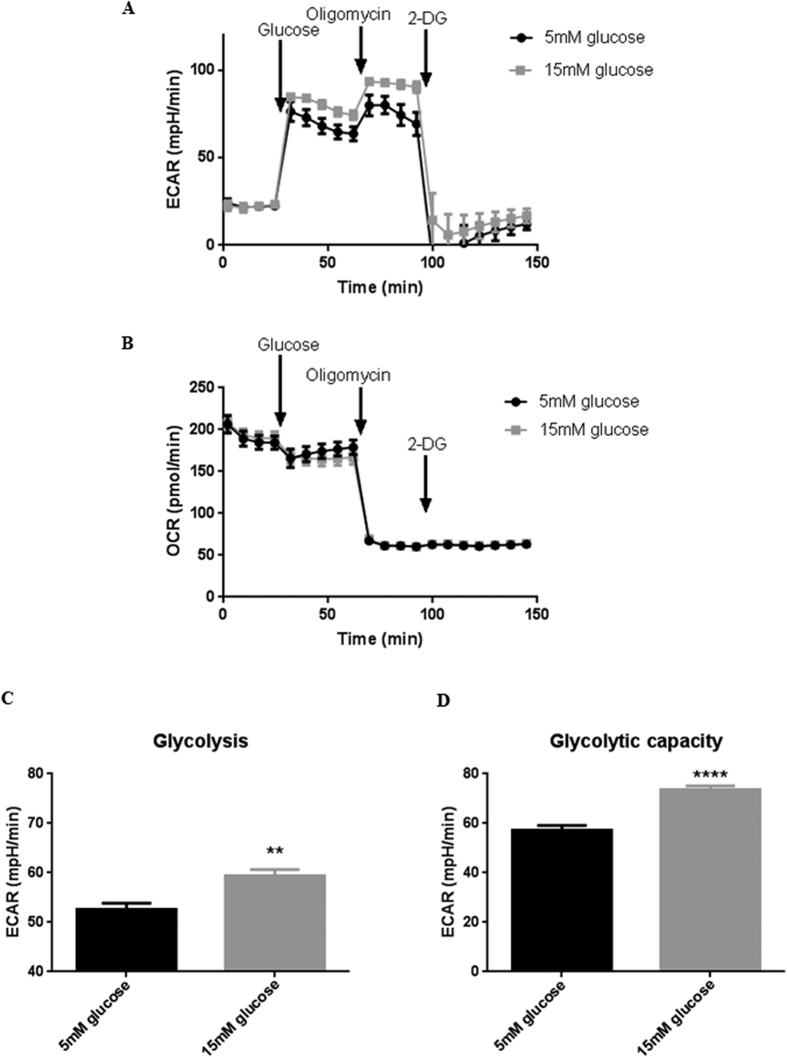
Hyperglycaemia induces a metabolic shift in airway epithelia towards aerobic glycolysis. (**A**) ECAR measurement trace and (**B**) OCAR measurement trace during Seahorse Glycolysis Stress Assay in which the medium bathing the Calu-3 cells was injected with of 5 or 15 mM Glucose, 1 μM Oligomycin and 75 mM Oligomycin. (**C**) Glycolysis rate, calculated by subtracting the normalized ECAR values after 2-Deoxy-D-Glucose (2-DG) injection from the ECAR values after glucose injection, in order to exclude the non-glycolytic acidification from the calculation. (**D**) Glycolytic capacity, calculated by subtracting the non-glycolytic acidification rate (ECAR after 2-DG injection) from the maximum ECAR after 1 μM Oligomycin injection. **P < 0.01, ****P < 0.0001, n = 12. Data represent the means ± S.E.M.

In order to study whether hyperglycaemia induced a shift in metabolism in airway epithelial cells, we measured glycolysis by extracellular acidification rate (ECAR; Fig. 2A) and respiration by oxygen consumption rate (OCR; Fig. 2B) in Calu-3 cells using a Seahorse Analyzer. Increasing the glucose content of the bathing medium from 5 to 15 mM had no significant effect on OCR, but led to a significant increase in the rate of aerobic glycolysis (Fig. 2C; P < 0.01; n = 12) and the glycolytic capacity of the Calu-3 cells (Fig. 2D; P < 0.0001; n = 12).

**Figure 3:**
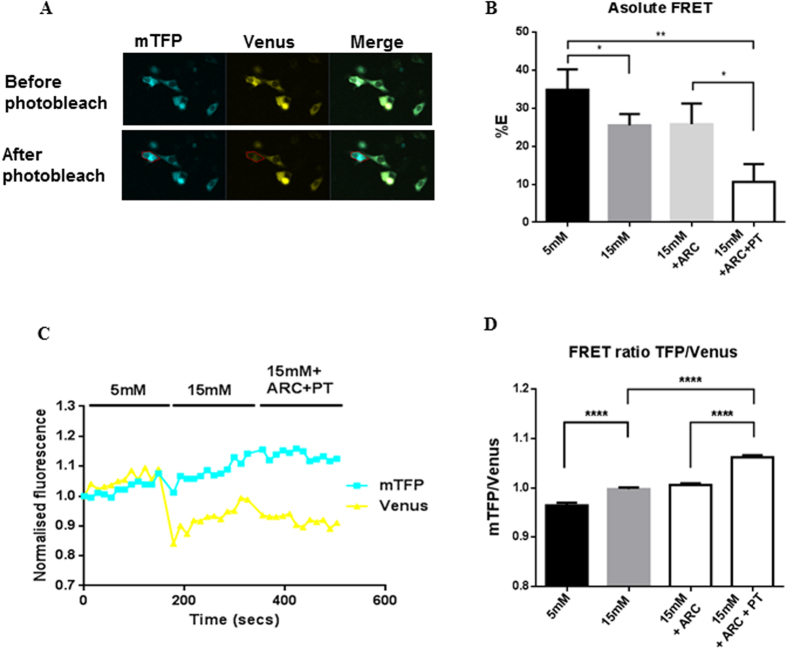
Hyperglycaemia induces an increase in intracellular lactate concentration. Changes in intracellular lactate concentrations induced by increasing extracellular D-glucose from 5 to 15 mM with and without 100 nM AR-C155858 (ARC) or 50 μM phloretin (PT) measured using a lactate FRET sensor (Laconic) expressed in H441 cells. (**A**) Fluorescence images of mTFP (blue), Venus (yellow) and merged images in cells before and after photobleach. (**B**) Calculated FRET efficiency (%E). (**C**) FRET ratio (mTFP/Venus) in an exemplar H441 cell, over time course of treatments. (**D**) Collated FRET ratio data *P < 0.05, **P < 0.01, ***P < 0.001, n = 24. Data represent the means ± S.E.M.

To confirm that hyperglycaemia increased intracellular lactate concentrations and thus an increased driving force for excretion via MCTs, H441 airway epithelial cells were transfected with the intracellular FRET sensor, Laconic^19^. Changes in intracellular lactate were analysed by independently measuring Laconic FRET efficiency and FRET ratio (Fig. 3A–D). Elevated basolateral glucose resulted in an increase in intracellular lactate, as evidenced by a decrease in FRET efficiency (P < 0.05; n = 24) and an increase in FRET ratio (P < 0.0001; n = 24).

### Inhibition of monocarboxylate transporters partially prevents hyperglycaemia-induced changes in ASL lactate concentration

Inhibition of monocarboxylate transporter MCT1/2 with AR-C155858 (100 nM) partially prevented the effects of hyperglycemia on ASL lactate (Fig. 1B–D; glucose-induced increase in lactate concentration reduced by 54% in HBE monolayers; by 47% in H441; by 54% in Calu-3; P < 0.05, n = 5–13) but did not reduce ASL lactate under normoglycemic conditions (data not shown). Phloretin (50 μM), a drug known to inhibit several MCTs had no additional effect on lactate concentration in the ASL (Fig. 1B).

Hyperglycaemia-induced increases in intracellular lactate in H441 cells could be further elevated by the addition of AR-C155858 (100 nM) + phloretin (50 μM) resulting in a 6.45% increase in FRET ratio (Fig. 3B; P < 0.0001; n = 24) and 14.91% decrease in FRET efficiency (Fig. 3D; P < 0.05; n = 24), consistent with inhibition of lactate efflux pathways.

### Airway epithelia express MCT2 and MCT4

**Figure 4:**
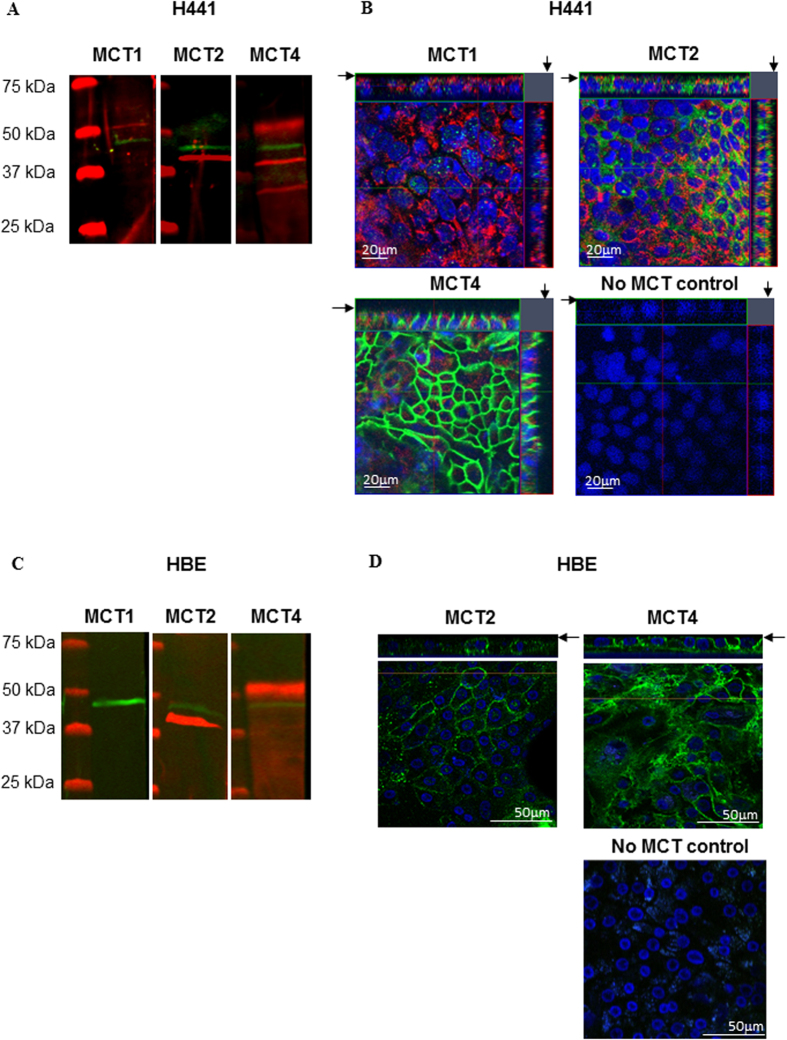
Airway epithelia express monocarboxylate transporters (MCT) 2 & 4. Western blot and confocal images of immunostained MCT in H441 (**A**,**B**) and HBE (**C**,**D**) cells grown on Transwell supports. (**A**,**C**) Western blots of MCT1 (not detected in HBE), 2 and 4 (red) and β-actin (green). (**B**,**D**) XY planes (centre) and XZ planes to the top and right (H441 only); apical surface indicated by an arrow; biotin-conjugated fluorophore labelling of the apical surface (red) (H441 only), MCT1, 2 and 4 (green) or absence of primary antiserum (no MCT control), DAPI stained nuclei (blue).

Immunostaining revealed no expression of MCT1 in primary HBE cells and little, predominantly intracellular staining for MCT1 in H441 cells (Fig. 4A–D). MCT2 was clearly immunostained in western blot as a ~40 kDa protein and was present in the cell membrane, including the apical membrane domain of both cell types. We did not observe any staining with antiserum to MCT3 in either cell type (data not shown). MCT4 western blot identified proteins of ~50, ~40, and ~30 kDa and there was clear basolateral localisation of MCT4 in H441 and HBE with limited expression in the apical membrane.

### Hyperglycaemia induces MCT-dependent ASL acidification in airway epithelial cultures lacking CFTR-dependent bicarbonate secretion

**Figure 5:**
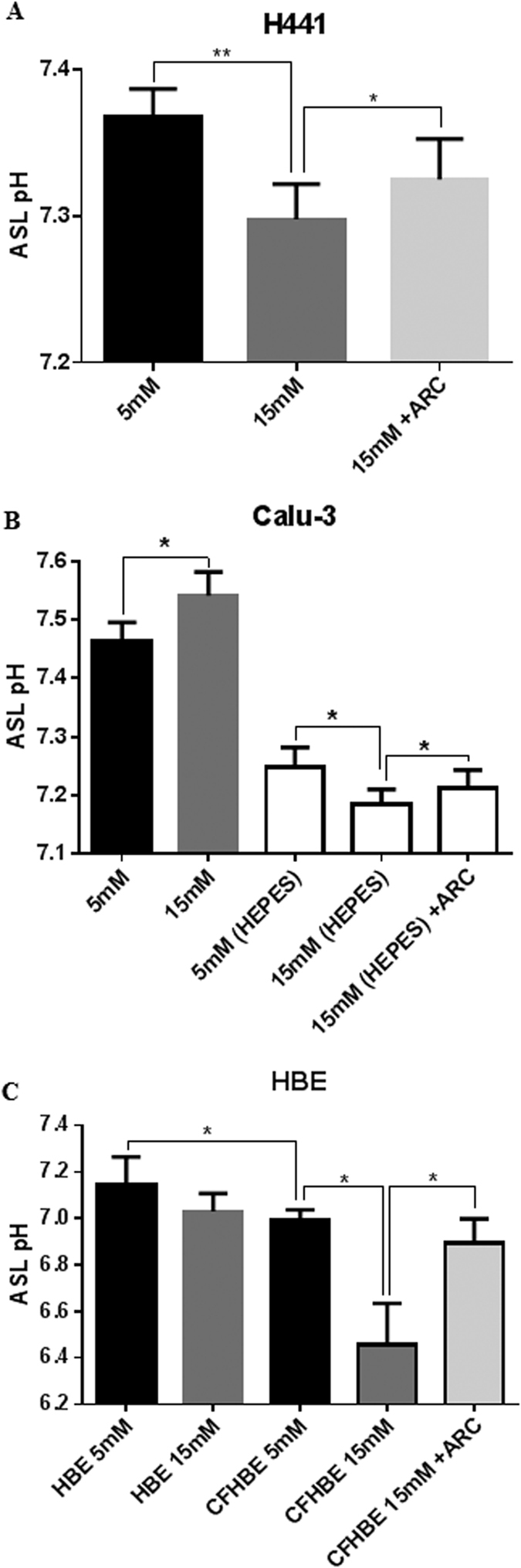
Hyperglycaemia induces MCT-dependent ASL acidification in airway epithelial cultures lacking CFTR-dependent bicarbonate secretion. (**A**) H441 ASL pH after 6 hours in 5 or 15 mM basolateral D-glucose, in the absence or presence of 100 nM MCT2 inhibitor AR-C155858 (ARC) *P < 0.05, **P < 0.01, n = 5. (**B**) Calu-3 ASL pH after 6 hours in HCO_3_^−^ or HEPES-buffered basolateral salt solution containing 5 or 15 mM D-glucose, in the absence or presence of 100 nM AR-C155858 (ARC) *P < 0.05, n = 9. (**C**) HBE or CF HBE ASL pH after 6 hours in 5 or 15 mM basolateral D-glucose, in the absence or presence of 100 nM AR-C155858 (ARC) *P < 0.05, n = 5. Data represent the means ± S.E.M.

As lactate is co-transported with H^+^, MCT-dependent lactate secretion would be predicted to acidify the ASL. In addition to increasing ASL lactate (Fig. 1), hyperglycemia also decreased ASL pH in H441 cells (Fig. 5A; P < 0.01; n = 5), but produced a slight alkalinisation in ASL of Calu-3 monolayers (Fig. 5B; P < 0.05; n = 9).

A key difference between Calu-3 and H441 epithelial cells is that Calu-3 cells possess CFTR and produce HCO_3_^−^ rich fluid secretions^16^. To test whether HCO_3_^−^ secretion in Calu-3 cultures compensated for lactate-driven H^+^ secretion, hyperglycaemia-induced changes in Calu-3 ASL pH were measured in cultures in which HCO_3_^−^ secretion was limited by removing HCO_3_^−^ from the bathing solution and replacing with HEPES. Under these conditions Calu-3 ASL acidified under hyperglycaemic conditions (Fig. 5B; P < 0.05; n = 9).

Hyperglycaemia-induced ASL acidification was also observed in primary HBE monolayers from CF patients (Fig. 5C; P < 0.05; n = 5), but not in non-CF HBE (Fig. 5C; P < 0.05; n = 5). Critically, inhibition of monocarboxylate transporter MCT2 with AR-C155858 (100 nM) significantly attenuated the hyperglycaemia-induced acidification of CF ASL.

### *Pseudomonas aeruginosa* enhances the hyperglycaemia-induced acidification of ASL pH across cystic fibrosis airway epithelial cultures

**Figure 6:**
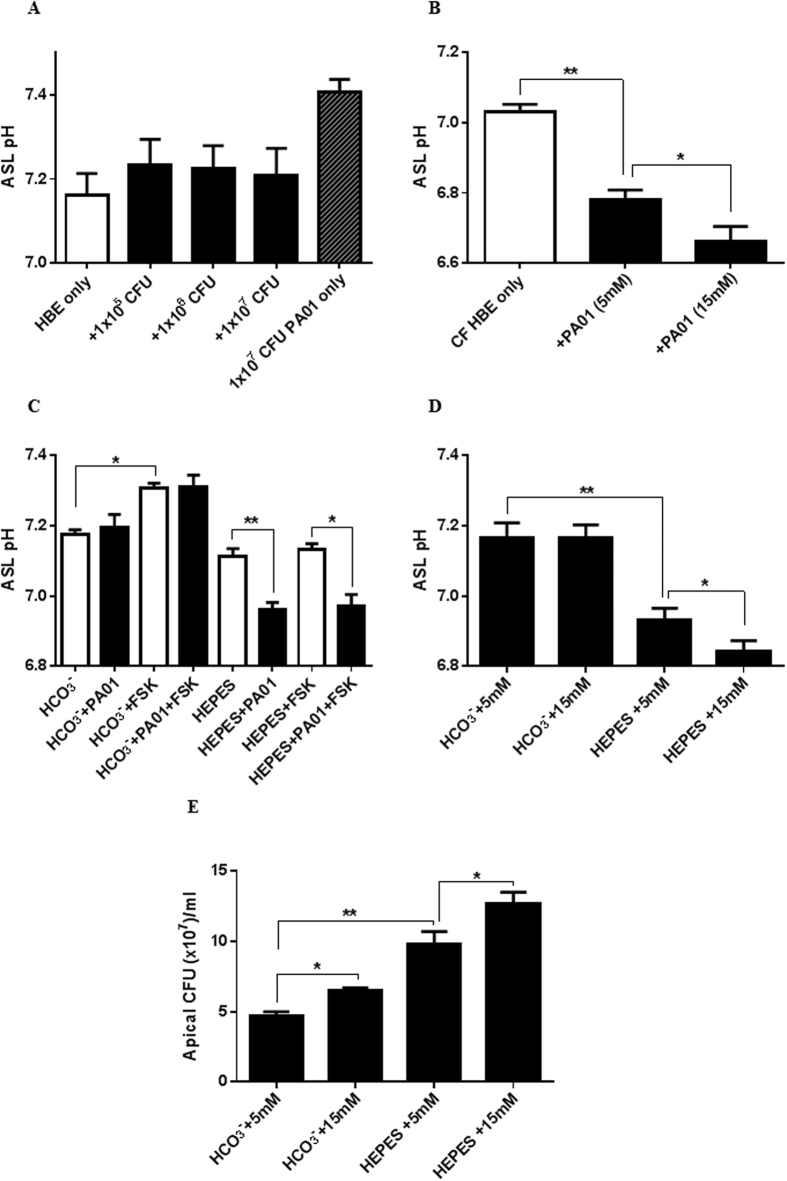
*P. aeruginosa* further acidifies airway ASL and enhances bacterial growth in the absence of CFTR-dependent bicarbonate secretion. (**A**) pH of HBE ASL after 6 hours in basolateral salt solution containing 5 mM D-glucose (HBE only) or in the presence of apical *P. aeruginosa* PA01 (+1 × 10^5^–10^7^ CFU), or pH of fluid from bacterial culture in the absence of HBE cells (1 × 10^7^ CFU PA01 only) n = 6. B. CF HBE ASL pH after 6 hours in the absence (CF HBE only) and presence of 1 × 10^6^ CFU apical PA01 (+PAO1), in basolateral HCO_3_^−^-buffered salt solution containing 5 or 15 mM D-glucose. (**C**) HBE ASL pH after 6 hours in the absence (open bars) or presence (black bars) of 1 × 10^6^ CFU apical PA01 (+PA01) in basolateral HCO_3_^−^ or HEPES-buffered salt solution containing 5 mM D-glucose with or without 5 μM forskolin (FSK) *P < 0.05, **P < 0.01, n = 5. D. HBE ASL pH after 6 hours in HCO_3_^−^ or HEPES-buffered basolateral salt solution containing 5 or 15 mM D-glucose in the presence of 1 × 10^6^ CFU apical PA01 *P < 0.05, **P < 0.01, n = 5. E. *P. aeruginosa* CFU on the apical surface of HBE monolayers after 6 hours in HCO_3_^−^ or HEPES-buffered basolateral salt solution containing 5 or 15 mM D-glucose *P < 0.05, **P < 0.01, n = 5. Data represent the means ± S.E.M.

Next we investigated how the airway epithelium responded to the addition of *P. aeruginosa* (PA01) and whether this response was altered in CF HBE cultures with and without hyperglycaemia. *P. aeruginosa* did not affect fluid pH in the absence of epithelial cells after 6 hours of growth (Fig. 6A; P > 0.05; n = 6; compared to solution pH = 7.4). The addition of *P. aeruginosa* (from 10^5^–10^7^ CFU) to the apical surface of non-CF HBE cells also had no effect on ASL pH (Fig. 6A; P > 0.05; n = 6). However, ASL from CF HBE cells became more acidic after the addition of 10^6^ CFU *P. aeruginosa* (decreased by 0.25 ± 0.02 pH units after 6 hours; Fig. 6B; P < 0.05; n = 5). This *P. aeruginosa*-induced acidification was enhanced by increasing basolateral glucose from 5 to 15 mM (42 ± 14% increase; Fig. 6B; P < 0.05; n = 5).

*P. aeruginosa*-induced ASL acidification could be replicated in non-CF HBE by replacing HCO_3_^−^ with a HEPES-buffered bathing solution to limit HCO_3_^−^ secretion. This was confirmed by the loss of forskolin-stimulated HCO_3_^−^ secretion and ASL alkalisation in HEPES buffered but not HCO_3_^−^ buffered cells (Fig. 6C). Increasing basolateral glucose also led to a greater ASL acidification of PA01 infected non-CF HBE cells in the presence of HEPES (Fig. 6D; P < 0.05; n = 5). Furthermore, replacing HCO_3_^−^ with HEPES increased *P. aeruginosa* growth/survival, which was further enhanced by raising the glucose concentration in the bathing solution (Fig. 6E; P < 0.01; n = 5).

**Figure 7:**
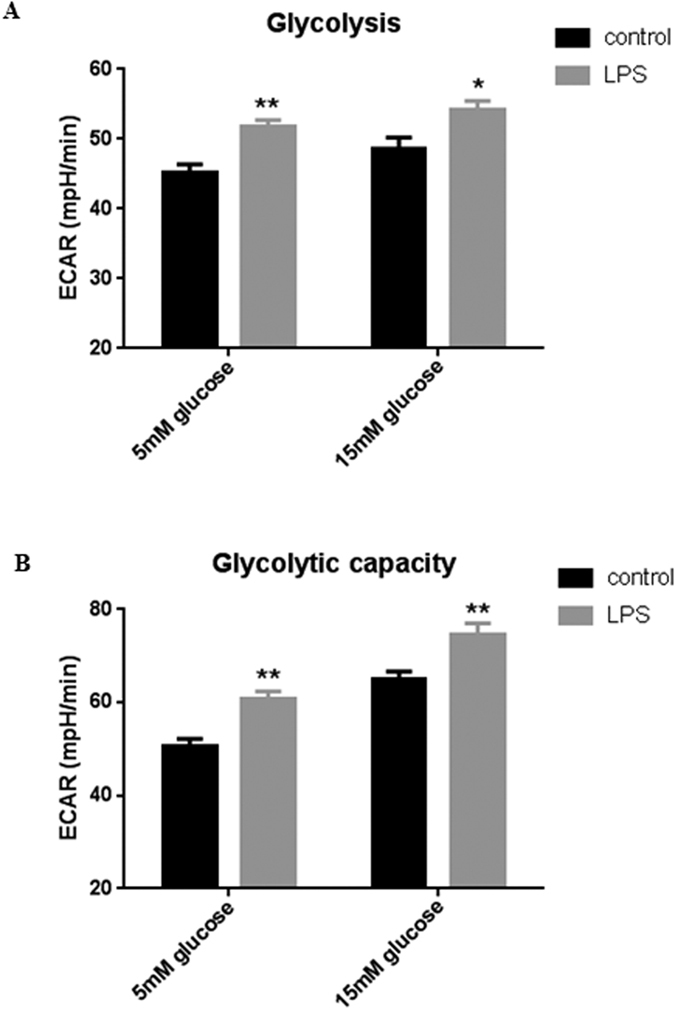
LPS increases the rate of aerobic glycolysis in airway epithelial cells. (**A**) Glycolysis rate of LPS-treated Calu-3 cells (24 hours pre-treatment), calculated by subtracting the normalized ECAR values after 2-Deoxy-D-Glucose (2-DG) injection from the ECAR values after glucose injection, in order to exclude the non-glycolytic acidification from the calculation. (**B**) Glycolytic capacity of LPS-treated Calu-3 cells (24 hours pre-treatment), calculated by subtracting the non-glycolytic acidification rate (ECAR after 2-DG injection) from the maximum ECAR after 1 μM Oligomycin injection. *P < 0.05, **P < 0.01, n = 11. Data represent the means ± S.E.M.

To investigate whether *P. aeruginosa* may induce changes in ASL pH through shifts in metabolism similar to those observed with hyperglycaemia, Calu-3 cells were exposed to LPS from *P. aeruginosa* and changes in metabolism (ECAR) were measured using a Seahorse Analyzer. LPS enhanced both aerobic glycolysis (Fig. 7A; P < 0.05; n = 11) and the glycolytic capacity (Fig. 7B; P < 0.01; n = 11) of the airway epithelial cells under normoglycaemic and hyperglycaemic conditions.

### *Pseudomonas aeruginosa*-induced changes in ASL lactate and pH across cystic fibrosis airway epithelial cultures can be overcome by MCT inhibition

**Figure 8:**
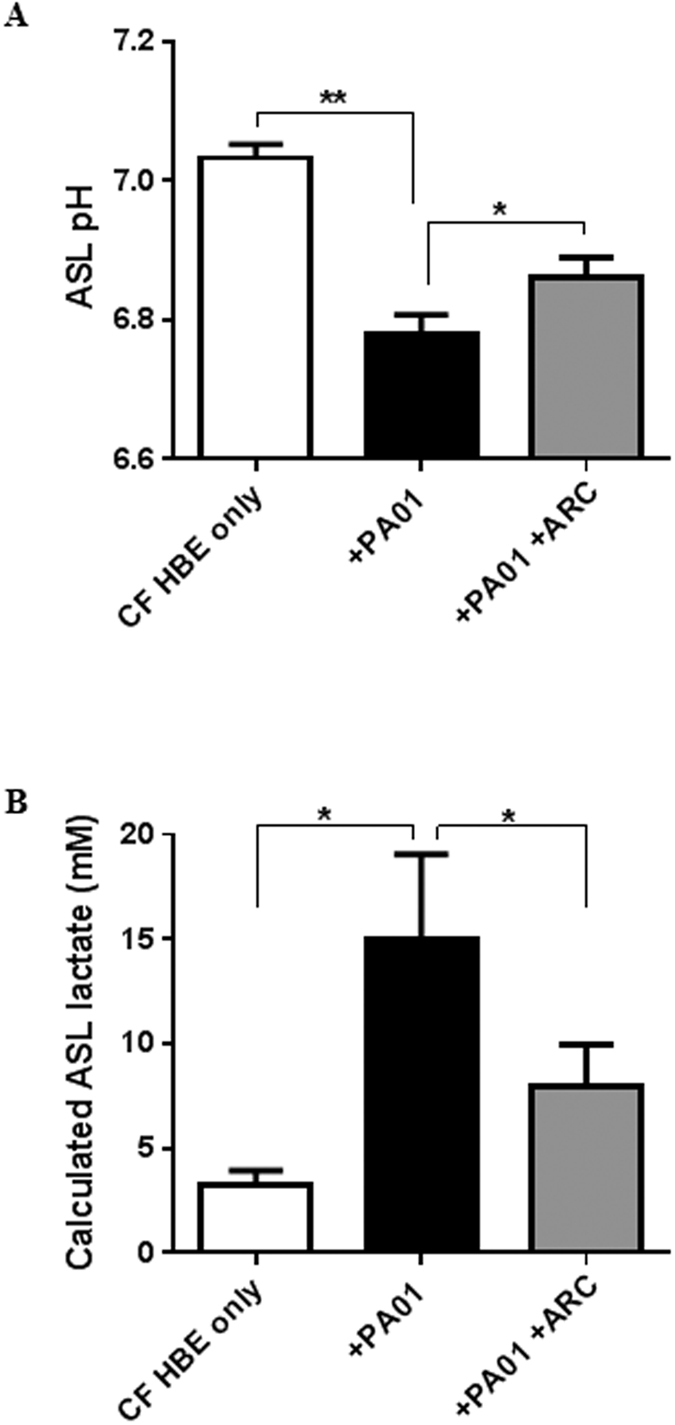
*P. aeruginosa* induces MCT-dependent elevation of lactate and ASL acidification in CFBE. (**A**) CF HBE ASL pH and G. ASL Lactate concentration after 6 hours in HCO_3_^−^-buffered basolateral salt solution containing 5 mM D-glucose in the absence (CF HBE only) or presence of 1 × 10^6^ CFU apical PA01 (+PA01), or 100 nM MCT2 inhibitor AR-C155858 (ARC). *P < 0.05, **P < 0.01, n = 5. Data represent the means ± S.E.M.

*P. aeruginosa*-induced acidification of CF ASL was attenuated in the presence of the MCT2 inhibitor AR-C155858 (Fig. 8A; P < 0.05; n = 5). PA01 did not produce lactate in isolated culture (data not shown), indicating that the bacterium induced these changes in CF ASL pH by stimulating epithelial lactate secretion. Consistent with this hypothesis, we found that ASL lactate production increased from 3.3 ± 0.7 mM to 14.9 ± 4.1 mM following the addition of *P. aeruginosa* (P < 0.05; n = 5), which could be significantly reduced by MCT2 inhibition (Fig. 8B; P < 0.05; n = 5).

## Discussion

Our data provide the first evidence that lactate is present in the ASL of human bronchial epithelial cells and that lactate concentration increased in ASL under hyperglycaemic culture conditions. We show that hyperglycaemia induced a metabolic shift towards aerobic glycolysis and increased intracellular lactate production. Our data also demonstrate a new role for MCT2 in mediating epithelial lactate efflux in airway cells in response to hyperglycaemia. During hyperglycaemic challenge of cells grown at air-liquid interface, apical application of the MCT1/2 specific inhibitor AR-C155858 reduced lactate in ASL but the general MCT inhibitor phloretin did not evoke any additional effect. In non-polarised cells phloretin but not AR-C155858 elevated intracellular lactate. Thus, these data point to the differential expression of transporters in polarised airway epithelial cells. In support of this notion, we demonstrated the presence of MCT2 on the apical surface and MCT4 on the basolateral surface of polarised epithelial cells. Thus, the apical localisation of MCT2 and action of AR-C155858 indicated that it mediated secretion of lactate into ASL. The basolateral localisation of MCT4 and lack of effect of apically applied PT in polarised cells (but not in non-polarised cells) indicated that MCT4 may sub serve another function. As lactate can also move across cell membranes via other pathways^21^, the incomplete inhibition of lactate secretion into the ASL by AR-C155858 would support other routes for lactate movement out of the cell and into ASL.

We did not detect MCT1 or MCT3 in human airway epithelial cells. MCT1 and 4 have previously been described in the developing rat and human airway^22^. However, the expression of MCT1 declined as the lungs developed from the pseudoglandular to saccular phase indicating that, consistent with our observations, MCT1 is unlikely to be present in adult cells^22^.

Mucosal addition of *P. aeruginosa* to airway epithelia increased epithelial lactate concentration in ASL. We previously demonstrated that airway epithelial glucose uptake was elevated in the presence of *P. aeruginosa and pro-inflammatory stimuli*^9^,^23^. Like hyperglycaemia (which increases the diffusion gradient for facilitative glucose uptake), increased glucose transport is also likely to drive a metabolic shift to increase cellular lactate production and secretion. We show that pro-inflammatory stimuli do indeed drive a metabolic shift towards enhanced aerobic glycolysis and increased lactate production. Moreover, increased uptake in the presence of hyperglycaemia would be expected to elevate lactate secretion even further, consistent with our results. *In vivo*, in the absence of, or prior to an inflammatory response, inducing such changes in host metabolism to elevate lactate in ASL may favour *P. aeruginosa* growth over other bacteria present in the airway microbiome as fewer species can utilise lactate as a growth source^24^. Moreover, these effects are likely to be exacerbated in the viscous mucus of the CF ASL^25^.

It is well established in CF that a lack of functional CFTR impairs airway HCO_3_^−^ and fluid secretion producing an acidic, viscous ASL^1^,^16^,^26^ that is readily colonised by bacteria such as *P. aeruginosa*. The acidity of the ASL leads to impaired activity of antimicrobial peptides and microbicidal effect in animal models of CF lung infections^2^. Our results indicate that the airway epithelium secretes HCO_3_^−^ which neutralises the acidifying effect of lactate secretion to maintain optimal ASL pH. Moreover, we show that this process is impaired in CF epithelium leading to acidification of ASL. Recently a transporter has been identified (ATP12A) which mediates H^+^ secretion in human airways but is absent in the mouse^27^. Our data indicate that acidification of ASL can also occur by MCT-driven H^+^ secretion when the epithelium is challenged with bacteria and/or during hyperglycaemia. Thus, these data further support the critical role of CFTR in the regulation of HCO_3_^−^ secretion and identify new key processes contributing to the acidic ASL of the CF lung.

Development of CFRD is associated with increased colonisation by *P. aeruginosa* and poorer outcomes^5^,^28^,^29^. We previously demonstrated that hyperglycaemia elevated ASL glucose and increased the growth/survival of *P. aeruginosa*^9^. Together with our current observations we propose that during CFRD, hyperglycaemia enhances bacterial colonisation of the ASL by providing a richer nutrient source (glucose and lactate) and a reduced pH which impair bacterial killing and favour growth. Our data are consistent with a critical role for CFTR-dependent bicarbonate secretion and ASL glucose homeostasis in airway innate immunity and the suppression of pathogen growth.

## Additional Information

**How to cite this article**: Garnett, J. P. *et al*. Hyperglycaemia and *Pseudomonas aeruginosa* acidify cystic fibrosis airway surface liquid by elevating epithelial monocarboxylate transporter 2 dependent lactate-H^+^ secretion. *Sci. Rep*. **6**, 37955; doi: 10.1038/srep37955 (2016).

**Publisher's note:** Springer Nature remains neutral with regard to jurisdictional claims in published maps and institutional affiliations.
